# Retarding mechanism of Zn^2+^ species in geopolymer material using Raman spectroscopy and DFT calculations

**DOI:** 10.1038/s41598-022-25552-0

**Published:** 2022-12-05

**Authors:** Fawzi Chamssine, Luiz H. S. Gasparotto, Miguel Angelo Fonsecade Souza, Mahmoud Khalifeh, Julio Cezar de Oliveira Freitas

**Affiliations:** 1 Department of Energy and Petroleum Engineering, Faculty of Science and Technology, University of Stavanger, 4036 Stavanger, Norway; 2 Universidade Federal do Rio Grande do Norte, UFRN, Natal, 59078-970 Brazil

**Keywords:** Materials chemistry, Chemistry, Materials science, Structural materials

## Abstract

Geopolymers are the most promising alternative to Ordinary Portland Cement for oil-well cementing and well abandonment. To that end, the slurry needs a required pumping time ensured by the addition of retarders. Although zinc has been widely known to prolong the setting time of geopolymers, its mechanism of action has yet to be fully elucidated. It is herein hypothesized that zinc ions impede the first stages of silicate oligomerization (Si–O–Al), culminating in longer setting times. Pumping time measurements showed that Zn(NO_3_)_2_ delayed the setting time by 5 h in comparison to the zinc-less sample. DFT calculations revealed Si(OH)_4_ to react with [Zn(OH)_4_]^2−^ via a barrierless transition state, evidencing a kinetic ground for the retardation effect. Additionally, Raman spectroscopy corroborated the DFT results by showing that Q^3^ species in the proposed mechanism are formed more rapidly in the presence of zinc ions than in its absence.

## Introduction

Geopolymers are an alternative cementitious material with the potential of replacing Ordinary Portland Cement (OPC) in both construction and oil & gas applications. The applicability of this material into oil and gas has been under research in the past period since its production has a lower carbon footprint and maintains superior properties over OPC specifically in long term periods^1–3^. However, to apply such material in cementing and well abandonment operations, chemical admixtures such as retarders must be used to delay setting and guaranty a safe period for displacement into wellbores^4^. Geopolymer formation from solid materials is a complex, multistep process roughly comprising i) alkaline depolymerization of the poly(siloxo) framework and dissolution of aluminum ii) formation of monomers and oligomers from ortho-sialate (OH)_3_^−^ Si–O–Al–(OH)_3_, and iii) polycondensation into higher oligomers and polymeric 3D networks^5,6^. Advantageously, it has been demonstrated that the degree of polymerization/depolymerization of glasses and geopolymers can be determined via Raman spectroscopy^7^. In essence, SiO_4_ species in a silica network differ from each other spectroscopically according to the number of sharing oxygen atoms. An isolated SiO_4_, for instance, is referred to as Q^0^ due to its lack of sharing oxygen. A Q^1^ entity denotes, in turn, a SiO_4_ with one sharing oxygen in the network. The reasoning extends then to Q^2^, Q^3^, and Q^4^ meaning two, three, and four sharing oxygen atoms, respectively. Upon contacting glass or a silica-rich mineral with an alkaline environment it is expected that the amount of Q^0^–Q^3^ species increase with time due to silica depolymerization, a phenomenon that can be tracked since each Q^n^ species appear at distinct frequencies in the Raman spectrum^7–9^.

Zinc (Zn^2+^) species, as a retarder, have been under study where its mechanistic and kinetic aspects have been taken into consideration^10–13^. Zinc oxide (ZnO), for instance, is thought to dissolve into Zn^2+^ which prolongs the setting time by sequestering calcium ions (Ca^2+^) and forming calcium zincate [Ca(Zn(OH)_3_)_2_.2H_2_O]^11^. This is also the conclusion reached by Cong et al.^14^, who only speculated that Zn^2+^ could have had an effect on the condensation polymerization. The possibility of Zn^2+^ playing a role in the early stages of the geopolymerization should not be overlooked. Zeng et al*.*^15^ demonstrated the synthesis of a coagulant based on poly-zinc-silicate to yield a complex compound with mainly zinc-silicon polymeric species rather than a simple mixture of raw materials. Upon studying the impact of Zn^2+^ and lead (Pb^+2^) ions on OPC, Oretgo et al*.*^16^ discovered Zn^2+^ ions to retard the silicate polymerization. The authors demonstrated, via NMR, a high proportion of Q^0^ and Q^1^ species after curing OCP with Zn^2+^ ions, implying a low degree of polymerization of SiO_4_ units.

The present study intends to shed light on the mechanism via which Zn^2+^ retards the geopolymerization reaction. The insight of this work resides in regarding the Zn^2+^, in the form of [Zn(OH)_4_]^2−^ due to the high pH, as a reactant that is inserted into monomers and oligomers to hamper momentarily the progression of the reaction. Density Functional Theory (DFT) calculations revealed that SiO_4_ reacts more quickly with [Zn(OH)_4_]^2−^ via a step with a barrierless transition state, meaning that the reaction between Si(OH)_4_ and [Zn(OH)_4_]^2−^ is kinetically more feasible in comparison to that between Si(OH)_4_ and Al[(OH)_4_]^−^. Raman results revealed that the presence of Zn^2+^ led to a higher rate of initial depolymerization of the poly(siloxo) framework, which supports the DFT proposition.

## Results and discussion

### Retardation phenomenon: granite-based geopolymer slurry

**Figure 1:**
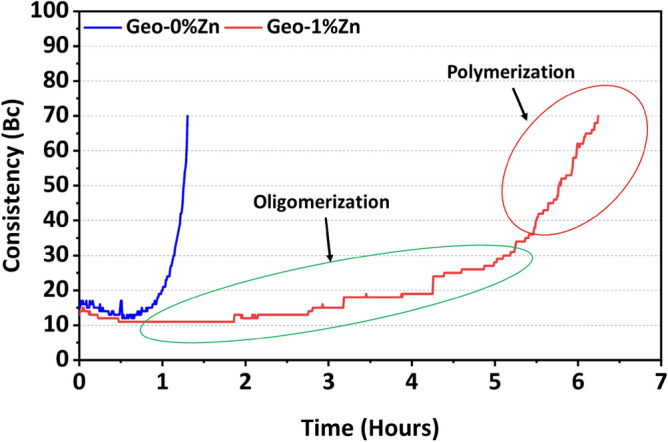
Effect of Zn^2+^ species on pumping time of granite-based geopolymer—oligomerization and polycondensation phases highlighted for Geo-1%Zn.

The impact of retardation phenomena in geopolymer material can be initially observed through pumping time measurements as described in previous studies by Chamssine et al*.*^1,4,17^. The retardation phenomenon can be observed through consistency measurements of slurries that show the behavior of material under different conditions. In this case, pumpability into an oil & gas well. The pumping time of the granite-based geopolymer slurries Geo-0%Zn and Geo-1%Zn is presented in Fig. 1. It can be observed that the addition of Zn^2+^ species (Geo-1%Zn) delays the pumping time by around 5 h in comparison to the neat sample (Geo-0%Zn), which reached its maximum after around 1 h. It can be perceived that the oligomerization phase was highly extended due to the presence of Zn^2+^ species. A poisoning phenomenon of the reaction can be concluded from Fig. 1, where the oligomerization and polycondensation phase was also affected. Previously, different reasonings have been given to explain behavior of Zn^2+^ in silica-rich geopolymer systems. Wang et al*.*^10^ analyzed the role of ZnO on metakaolin based geopolymer material. They concluded that the presence of ZnO have created metastable “Na/K-Zn” phase materials (Na^+^ and K^+^ sourced from the alkali activator), which have a prolonging effect on setting time. Their study also included the investigation of Q^4^(3Al) and Q^4^(4Al) sites using NMR spectra. A decrease in the Si/Al ratio was observed due to the increase in number of Q^4^(4Al) sites. It has been foreseen that not only Q^4^ sites to be affected, but rather also the possibility of having a deviation in Q^3^ sites’ development, which can as well be sites to investigate while the reaction is developing^18^.

### Computational model

The calculated *Gibbs* free energy of reaction (Δ_r_G) for fifteen dehydration reactions involving the Si(OH)_4_, [Al(OH_4_)]^−^, [Zn(OH_3_)]^−^, and [Zn(OH_4_)]^2−^ monomers (and also some oligomers) are presented in Table 1. The condensation reaction (reaction 1) between Si(OH)_4_ and [Al(OH_4_)]^−^ monomers is exergonic by 11.0 kcal mol^−1^. Besides, condensation reactions to yield the (OH)_2_Al–(O–Si(OH)_3_)_2_, (OH)Al–(O–Si(OH)_3_)_3_, and Al–(O–Si(OH)_3_)_4_ oligomers are exergonic by 20.4, 30.5, and 38.7 kcal mol^−1^, respectively (reactions 2–4 in Table 1). These results suggest that the formation of the Al–O–Si linkage at the [(OH)_3_Al–O–Si(OH)_3_]^−^ (*ortho*-sialate) and oligomers species, which is the driving force of the reaction, is in line with the explanation previously given that the geopolymerization mechanism must occur through the condensation of oligomers^19^.

**Table 1:** *Gibbs* free energy of reaction (Δ_r_G. in kcal mol^−1^) computed at the ωB97X-D/6–311 + G(3df.2p)//6–31 + G(d.p) level of theory.

		Δ_r_G
	**Condensation**	
(1)	[Al(OH)_4_]^−^ + Si(OH)_4_ → [(OH)_3_Al–O–Si(OH)_3_]^−^ + H_2_O	–11.0
(2)	[Al(OH)_4_]^−^ + 2Si(OH)_4_ → [(OH)_2_Al–(O–Si(OH)_3_)_2_]^−^ + 2H_2_O	–20.4
(3)	[Al(OH)_4_]^−^ + 3Si(OH)_4_ → [(OH)Al–(O–Si(OH)_3_)_3_]^−^ + 3H_2_O	–30.5
(4)	[Al(OH)_4_]^−^ + 4Si(OH)_4_ → [Al–(O–Si(OH)_3_)_4_]^−^ + 4H_2_O	–38.7
(5)	[Zn(OH)_3_]^−^ + Si(OH)_4_ → [(OH)_2_Zn–O–Si(OH)_3_]^−^ + H_2_O	–7.5
(6)	[Zn(OH)_3_]^−^ + 2Si(OH)_4_ → [(OH)Zn–(O–Si(OH)_3_)_2_]^−^ + 2H_2_O	–18.2
(7)	[Zn(OH)_3_]^−^ + 3Si(OH)_4_ → [Zn–(O–Si(OH)_3_)_3_]^−^ + 3H_2_O	–26.9
(8)	[Zn(OH)_4_]^2−^ + Si(OH)_4_ → [(OH)_3_Zn–O–Si(OH)_3_]^2−^ + H_2_O	–18.9
(9)	[Zn(OH)_4_]^2−^ + 2Si(OH)_4_ → [(OH)_2_Zn–(O–Si(OH)_3_)_2_]^2−^ + 2H_2_O	–36.0
	**Dehydration**	
(10)	[Al(OH)_4_]^−^ + Si(OH)_4_ → [Al(OH)_3_] + [Si(OH)_3_O]^−^ + H_2_O	+ 35.0
(11)	[Zn(OH)_3_]^−^ + Si(OH)_4_ → [Zn(OH)_2_] + [Si(OH)_3_O]^−^ + H_2_O	+ 1.5
(12)	[Zn(OH)_4_]^2−^ + Si(OH)_4_ → [Zn(OH)_3_]^−^ + [Si(OH)_3_O]^−^ + H_2_O	–13.3
(13)	[Al(OH)_4_]^−^ + [(OH)_3_Al–O–Si(OH)_3_]^−^ → [Al(OH)_3_] + [(OH)_3_Al–O–Si(OH)_2_O]^2−^ + H_2_O	+ 44.5
(14)	[Zn(OH)_3_]^−^ + [(OH)_3_Al–O–Si(OH)_3_]^−^ → [Zn(OH)_2_] + [(OH)_3_Al–O–Si(OH)_2_O]^2−^ + H_2_O	+ 11.0
(15)	[Zn(OH)_4_]^2−^ + [(OH)_3_Al–O–Si(OH)_3_]^−^ → [Zn(OH)_3_]^−^ + [(OH)_3_Al–O–Si(OH)_2_O]^2−^ + H_2_O	–3.8

For the oligomers is being considered the condensation from 1 to 4 Si(OH)_4_ species.

**Figure 2:**
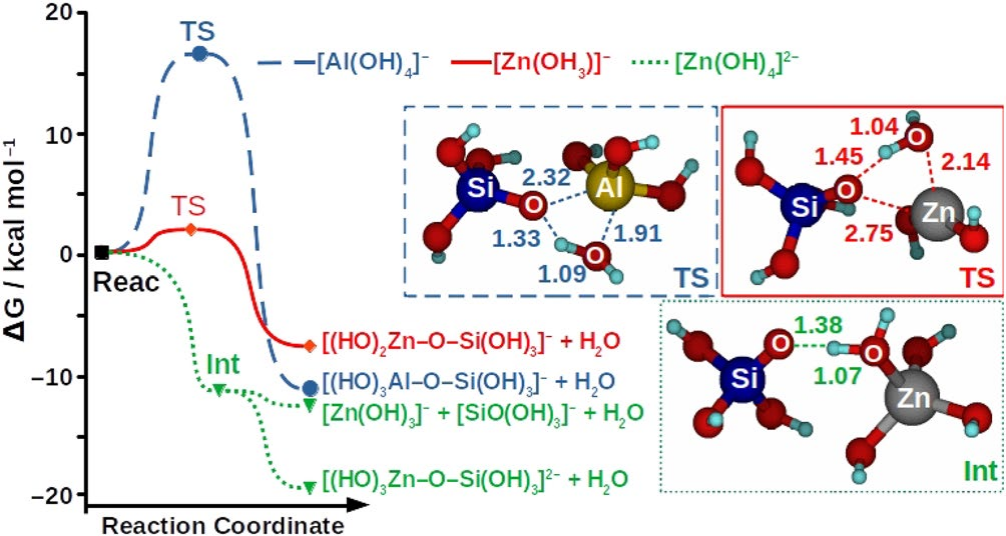
Left: *Gibbs* energy profiles calculated at the ωB97X-D/6–311 + G(3df.2p)//6–31 + G(d.p) method for the [Al(OH)_4_]^−^  + Si(OH)_4_. [Zn(OH)_3_]^−^  + Si(OH)_4_. and [Zn(OH)_4_]^2−^  + Si(OH)_4_ reactions. The *Gibbs* energy values (in kcal mol^−^) for each reaction profile are relative to the reactants (Reac). Structures of the transition states (TS) and intermediate (Int) calculated at the ωB97X-D/6–31 + G(d.p) level of theory. Some relevant bond distances (in Å) are included in the structures.

The calculations also predict the formation of the Zn–O–Si linkage which is favorable in terms of the *Gibbs* energy. Condensation reactions from the [Zn(OH_3_)]^−^ and [Zn(OH_4_)]^2−^ species are all exergonic (reactions 5–9), suggesting that Zn–O–Si units can be incorporated into the polymeric framework. However, the dehydration reactions without the condensation of the reactant species are only favorable for reactions involving the [Zn(OH_4_)]^2−^ specie (reactions 10–15). Indeed, the reactions 12 and 15 are computed to be exergonic by 13.3 and 3.8 kcal mol^−1^. Figure 2 depicts the *Gibbs* energy profiles and the relevant optimized structures calculated for the [Al(OH_4_)] ^−^  + Si(OH)_4_, [Zn(OH_3_)] ^−^  + Si(OH)_4_, and [Zn(OH_4_)]^2−^  + Si(OH)_4_ reactions. In our model, the condensation reactions from the [Al(OH_4_)]^−^ and [Zn(OH_3_)]^−^ anionic species are described by mechanism analogous. The reactants are linked to each other by the attraction between hydrogens from the Si(OH)_4_ specie and OH groups from the ionic specie, [Al(OH_4_)]^−^ or [Zn(OH_3_)]^−^. Then, the two reactants condense to form the [(OH)_3_Al–O–Si(OH)_3_]^−^ and [(OH)_2_Zn–O–Si(OH)_3_]^−^ species, respectively, with concomitant to release the H_2_O molecule. Based on Fig. 2, it should be pointed out that the [Al(OH_4_)]^−^  + Si(OH)_4_ and [Zn(OH_3_)]^−^  + Si(OH)_4_ reactions proceed through a transition state (TS) located at 16.4 and 2.1 kcal mol^−1^ above the reactants’ energy, respectively. The origin of the difference between these reaction barriers can be attributed to steric hindrance at the TS structures. As shown in Fig. 2, the TS of the reaction with the [Al(OH_4_)]^−^ specie involves a greater geometrical deformation when compared to the TS of the reaction with [Zn(OH_3_)]^−^.

On the contrary to what is computed for the Si(OH)_4_ + [Al(OH_4_)]^−^/[Zn(OH_3_)]^−^ reactions, the mechanism of the reaction between [Zn(OH_4_)]^2−^ and Si(OH)_4_ species is predicted to proceed by the initial formation of the [Zn(OH_3_)(OH_2_)…OSi(OH)_3_]^2−^ intermediate (Int). The formation of the Int occurs virtually barrierless, where the Si(OH)_4_ specie undergoes a hydrogen abstraction process by the OH group from the [Zn(OH_4_)]^2−^ (Fig. 2). Then, the Int can either condense to form the [(OH)_4_Zn–O–Si(OH)_3_]^2−^  + H_2_O products or fragment to yield the [Zn(OH)_3_]^−^  + [SiO(OH)_3_]^−^  + H_2_O products. According to our calculations, the reactions involving the [Zn(OH_4_)]^2−^ anions are kinetically and thermodynamically more feasible than that reaction with other anions.

### Raman spectroscopy

**Figure 3:**
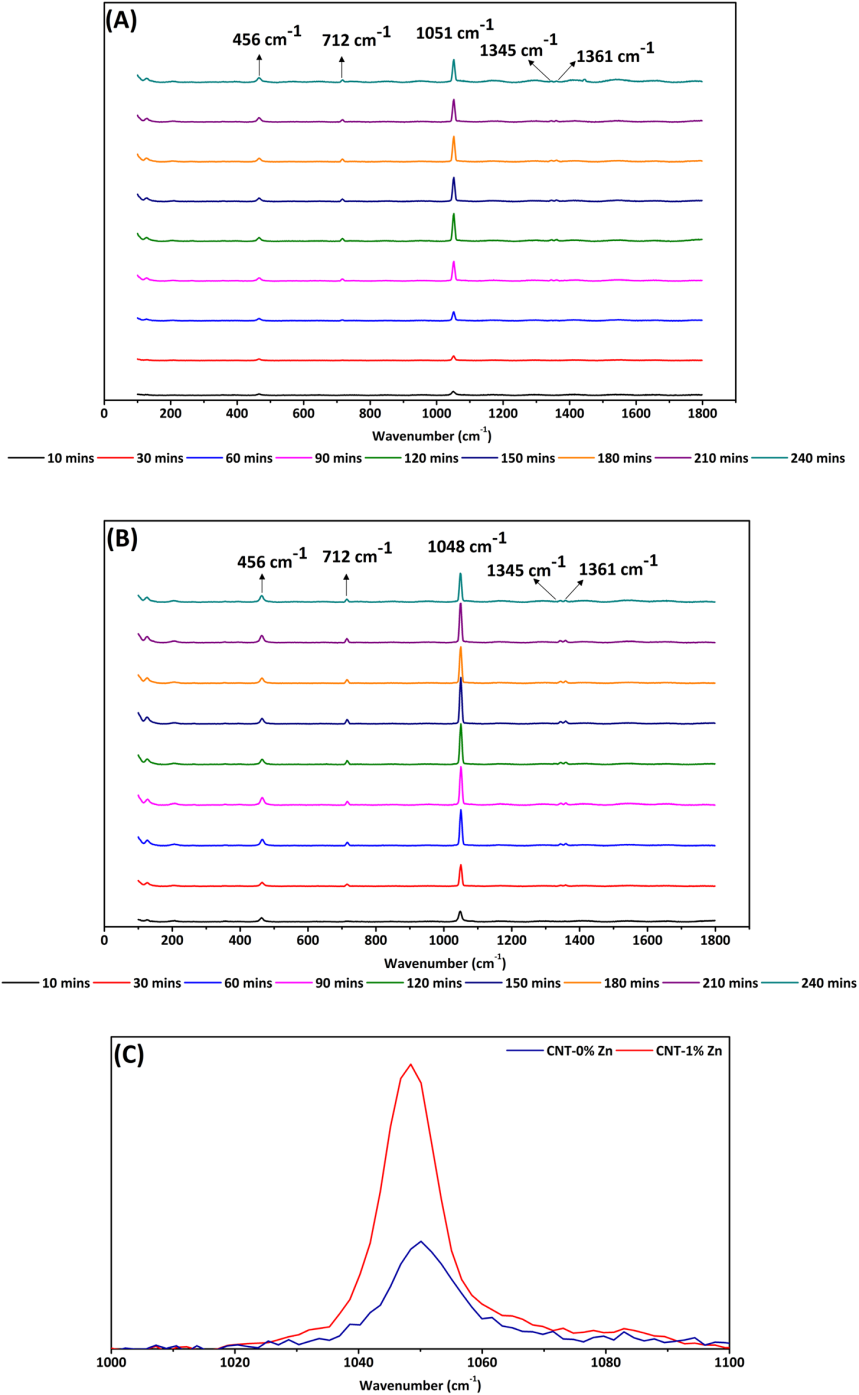
(**A**) Raman spectra of CNT-0%Zn; (**B**) Raman spectra of CNT-0%Zn; (**C**) Overlay of Raman spectra from geopolymer pastes having CNT-0%Zn and CNT-1%Zn (t = 10 min).

**Figure 4:**
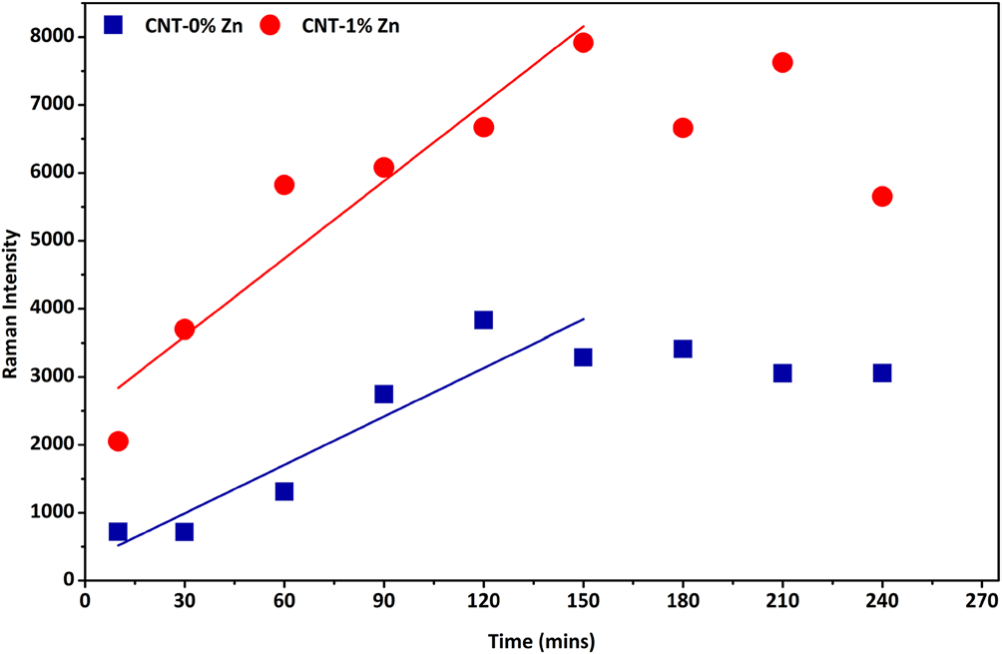
Q^3^ band intensity as a function of time for geopolymer pastes CNT-0%Zn & CNT-1%Zn.

Raman spectroscopy has proven to be a valuable tool in the study of geopolymers^8,9^. An advantageous feature is that their Raman spectra can be compared with those of SiO_4_ glasses^7^. SiO_4_ glass creation relies on the condensation of isolated SiO_4_ tetrahedra (referred to as Q^0^) by linking to each other via sharing one to four oxygen atoms (Q^1^–Q^4^). Since differently bonded tetrahedra have distinct Raman signatures in the range of 1000 cm^−1^–1100 cm^−1^, variations in that region may be used to track the SiO_4_ depolymerization required for geopolymer formation^7^. Interestingly, the computational chemistry results in Fig. 2 hints to the formation of Q^3^ species via a barrierless TS that appears only in the presence of Zn^2+^. It is then conceivable that Zn^2+^ would lead to a higher rate of Q^3^ formation in geopolymers when compared to their no Zn counterparts (CNT-0%Zn), which would support the mechanism determined by computational chemistry. This hypothesis has been verified by means of Raman spectroscopy with results shown in Fig. 3. Typical bands of quartz (silica flour) are probed at initial stages of geopolymerization (without Zn^2+^ at this point): 207 cm^−1^ (Si–O-Si bond twisting), 355 cm^−1^ (SiO_4_ bending), and 456 cm^−1^ (bending of O-Si–O)^20^, which tend to vanish due to quartz consumption. Vibrations at 712 cm^−1^, 1345 cm^−1^, and 1361 cm^−1^ refer to CO_3_^−2^ in calcite as a consequence of natural carbonation^21^, which is known to take place in cementitious materials such as OPC^22^. Regarding geopolymers, the reaction between CO_2_ from the air and OH^−^ produces Ca and sodium (Na) carbonates^23,24^. It is important to notice that the bands assigned to carbonate display no clear tendency over time, which is expected given that the variable amount of CO_2_ in the atmosphere leads to an uncontrolled carbonation process. The most important feature of Fig. 3A is the sharp peak at 1051 cm^−1^ related to a Si–O- vibration with O^−^ denoting a non-bridging oxygen within a Q^3^ species^8,25^. Another important characteristic is that the Q^3^ band initially increases with time (up to 150 min), meaning that OH^−^ attacks the Si network to produce increasing amounts of unbounded oxygen. It is important to emphasize that the increase in intensity of the peak at 1051 cm^−1^ is not a result of any artifact since; i) the time of laser illumination and laser potency were the same for all samples (ruling out intensity variation due to heating effects), ii) the illuminated spot of the sample was always the same (same area probed), and iii) there is no contribution of carbonation in the wavenumber region of interest^25^. Upon addition of 1% of Zn to the mixture (Fig. 3B), the respective overlaying Raman spectrum in Fig. 3C reveals a clear shift towards lower frequencies (now centered at 1048 cm^−1^) as a consequence of weakened Si–O force due to charge transfer from Zn(OH)_4_^2−^ to SiO_4_. The negative charge transferred to tetrahedra accumulates preferentially on Si atoms leading to a decrease in the Si–O coulombic interaction^26^. When comparing Q^3^ peaks, Fig. 4 clearly attests that the addition of 1% of Zn culminates in a higher rate of Q^3^ formation. The slopes of the straight lines from t = 0 to t = 150 min are 23.75 and 37.97 for mixtures without and with Zn^2+^, respectively. Both curves reach the steady state at 150 min, a situation in which the rate of Q^3^ formation is counterbalanced by the emergence of oligomers and subsequently geopolymer.

## Materials and methods

### Material and mixing process

A granite-based aluminosilicate-rich solid precursor has been used to exemplify the retardation effect of Zn^2+^. The precursor was designed with a low Ca content composition (< 10 wt.%) as presented in Table 2. A potassium silicate solution with molar ratio of 2.21 was used as an activating hardener. Zinc nitrate hexahydrate (Zn(NO_3_)_2_0.6H_2_O) was used as the source of Zn^2+^ species. The mix design of slurries is presented in Table 3.

**Table 2:** Composition of the granite-based geopolymer.

Chemical element	wt.%
SiO_2_	63.10
Al_2_O_3_	12.97
Fe_2_O_3_	1.49
CaO	9.94
MgO	4.54
Na_2_O	2.34
K_2_O	3.81
TiO_2_	0.80
MnO	0.19
LOI	0.80
Total	100

**Table 3:** Granite-based mix designs examined for pumping time.

Mix design components (Wt%)				
Mix Design	Solid	Liquid	Zn(NO_3_)_2_.6H_2_O Wt%	Number of molecules of Zn^2+^
Geo-0%Zn	66.23	53.77	-	-
Geo-1%Zn	66.23	53.77	1	1.21 × 10^+22^

### Control system (CNT): lab scale chemical components

In this study, a controlled system has been constructed using pure components that can replace certain compositions from the original granite-based precursor (presented in Table 2). The components have a purity of over 90%. The composition of the solid phase of the controlled system (CNT) is presented in Table 4, and list of chemical replacements used is presented in Table 5. The purpose behind using such a system is to isolate complex minerals and create a controlled system where the reaction progression can be monitored throughout time. Zn^2+^ species, in the form of Zn(NO_3_)_2_0.6H_2_O, were also used to mimic retardation with a similar 1 wt% as in the original mix design. In order to accurately mimic the designated components’ behavior, the number of molecules of Si, Al, Fe, Ca, Mg, Na, and K was calculated and implemented with the proposed chemical replacements^27^ (Table 5).

**Table 4:** Composition considered for controlled system under study derived from original precursor (Table 2).

To be considered in the reactants’ composition								Not to be considered due to low concentrations					
Composition	SiO_2_	Al_2_O_3_	Fe_2_O_3_	CaO	MgO	Na_2_O	K_2_O	TiO_2_	MnO	SrO	BaO	LOI	Total
W%	63	13	1.5	10	4.5	2.34	3.81	0.8	0.19	0.01	0.01	0.72	100

**Table 5:** List of composition, chemical replacement, and number of molecules for each replaced element.

Component in compositions	Chemical replacement	Chemical formula	Number of molecules
Si	Silica flour	SiO_2_	4.423 × 10^+24^
Al	Aluminum Nitrate Nanohydrate	Al(NO_3_)_3_·9H_2_O	5.358 × 10^+23^
Fe	Iron (II) Sulfate	FeSO_4_	3.94 × 10^+22^
Ca	Calcium Hydroxide	Ca(OH)_2_	7.47 × 10^+23^
Mg	Magnesium Oxide	MgO	4.75 × 10^+23^
Na	Sodium Hydroxide	NaOH	1.59 × 10^+23^
K	Potassium Hydroxide	KOH	1.71 × 10^+23^
Zn	Zinc Nitrate Hexhydrate	Zn(NO_3_)_2_.6H_2_O	1.21 × 10^+22^

Two samples were developed, CNT-0%Zn & CNT-1%Zn, where the former contains no Zn^2+^ species while the latter contains 1wt% of Zn. The composition of CNT-0%Zn & CNT-1%Zn solid phases are mentioned in Table 6, while the total mix designs are presented in Table 7. The alkaline hardener phase, a 4 M potassium hydroxide (KOH) solution, was produced with KOH laboratory grade pellets and distilled water. The use of KOH in this system was implemented to avoid polycondensation and coagulants where it can start to form instantly after introduction of potassium silicate solution to the solid phase with free Ca^2+^ species. This phenomenon was reported in a study by Nachbaur et al*.*^28^ where authors examined the electrokinetic properties which interfere with the suspension of silicates in early age hydration. They concluded that the presence of high Ca content in the composition can cause coagulation of Ca_3_SiO_5_ particles due to the low zeta potential under these conditions.

**Table 6:** Solid phase composition of controlled samples (CNT).

Components (Purity > 90%)	Mix design components (g)	
	CNT-0%Zn	CNT-1%Zn
SiO_2_	44.13	44.13
Al(NO_3_)_3_·9H_2_O	18.96	18.96
FeSO_4_	0.99	0.99
Ca(OH)_2_	9.19	9.19
MgO	3.18	3.18
NaOH	1.06	1.06
KOH	1.59	1.59
Zn(NO_3_)_2_.6H_2_O	–	0.79
Total	79.1	79.89

**Table 7:** Mix design of control samples.

Mix design components (Wt%)				
Mix design	Solid	Liquid	Zn(NO_3_)_2_.6H_2_O	Number of molecules of Zn^2+^
CNT-0%Zn	66.6	33.3	–	–
CNT-1%Zn	66.6	33.3	1	1.21 × 10^22^

Samples were prepared by dry mixing of the solid components first followed by the addition of alkaline solution (4 M KOH). The solid-to-liquid ratio was around 2.0 (Table 7). Initially, hand mixing was applied for 2 min (mins), then the apparatus was moved to a Hamilton Beach mixer with a single spindle for 60 s. The material had a honey-like consistency at the end of mixing.

### Testing and characterization methods

#### Consistency Measurements

An OFITE HPHT consistometer (Model 2040) was used to examine the pumping and setting time at a BHCT of 50 °C and pressure of 2000 psi. The standard for pumping time was set from the starting point until 40 BC while setting time was from 40 to 100 Bc following API RP 10-B2 recommendations^29^. This test was performed only on granite-based geopolymer slurry.

#### *Raman* Spectroscopy

Geopolymer paste, of the controlled system samples (CNT), was analyzed using Raman Spectroscopy. Spectra from 400 to 4000 cm^−1^ were examined using a LabRAM HR Evolution using a 532 nm diode laser operating at 25 mW. Spectra were collected at 30 min time interval. Freshly mixed geopolymer paste was analyzed at time (t) 0 and transported to a nearby oven operating at 50 °C. Thus, maintaining the reaction temperature like the one in the consistometer.

### Computational chemistry model description

**Figure 5:**
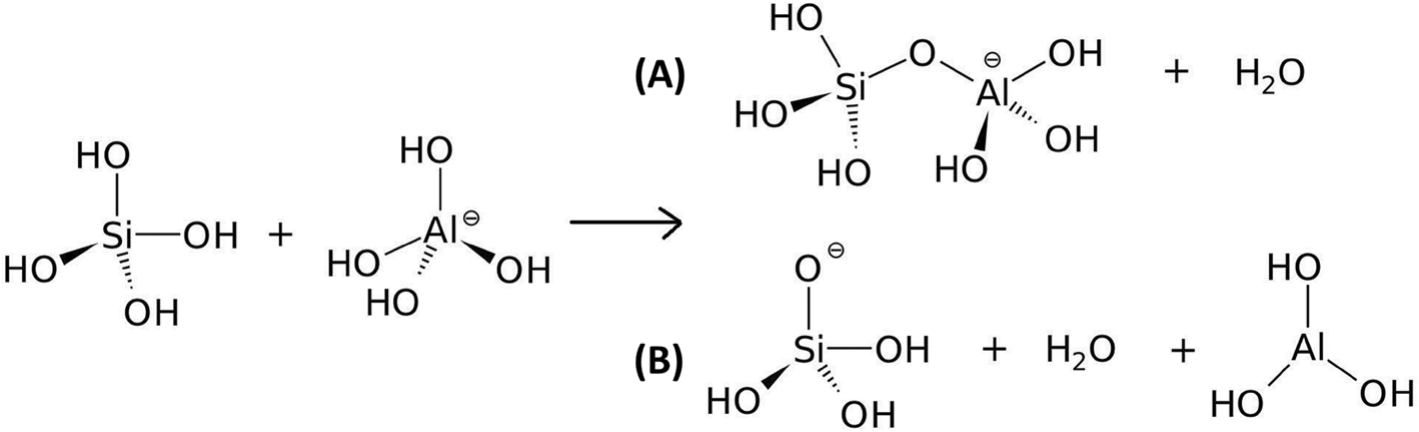
Dehydration reactions (**A**) with and (**B**) without condensation of the reactant species.

Density functional theory (DFT) calculations were performed to support the experimental evidence of the retardation effect of Zn^2+^ in the geopolymerization reaction following the method presented by Yang et al*.*^30^. For such, dehydration reactions between the Si(OH)_4_ and [Al(OH)_4_]^−^, [Zn(OH)_3_]^−^ and [Zn(OH)_4_]^2−^ species were used to model the formation of the Al–O–Si and Zn–O–Si bonds^31^. In our model, Si, Al, and Zn are coordinated by hydroxides, which is consistent with the high alkaline condition (pH around 13.0 to 13.5) used in the experimental setup^32^. In this range of pH, there is an equilibrium between [Zn(OH)_3_]^−^ and [Zn(OH)_4_]^2−^ anionic species. Figure 5 presents two types of dehydration reactions considered in this study:

First, structure predictions of the reactants and products were performed with the conformer-rotamer ensemble sampling tool of the xtb software^33,34^. To globally explore conformers, the GFN2-xTB method was used in the framework of meta-dynamics^35,36^. Secondly, the minimum-energy conformers were chosen as guess structures for calculations of geometry optimizations with the ωB97X-D/6-311 + G(3df.2p)//6-31 + G(d.p) level of theory. The structures were optimized at the ωB97X-D/6-31 + G(d.p) level of theory, and single-point calculations were performed at the ωB97X-D/6-311 + G(3df.2p) on these structures. All calculations were performed with an implicit solvent (water) using the polarizable continuum model. The ωB97X-D functional has presented a good performance in the description of structural kinetics and thermochemical properties^37,38^. The Gaussian 16 software package was used for all DFT calculations^39^.

## Data Availability

The datasets generated during the current study are available from the corresponding author upon request.
